# Efficacy of convalescent plasma in hospitalized COVID-19 patients: findings from a controlled trial

**DOI:** 10.1590/1414-431X2024e13627

**Published:** 2024-10-07

**Authors:** T.P. Costa, M. Aoki, C.M. Ribeiro, E. Socca, L. Itinose, R. Basso, L. Blanes

**Affiliations:** 1 Diretoria Técnica e Qualidade, Maternidade e Cirurgia Nossa Senhora do Rocio-HR, Campo Largo, PR, Brasil; 2 Laboratório de Ciência e Tecnologia Aplicada è Saúde, Instituto Carlos Chagas, Fundação Oswaldo Cruz, Curitiba, PR, Brasil; 3 Instituto de Pesquisa do Vale da Ciência, São Paulo, SP, Brasil

**Keywords:** COVID-19, Convalescent plasma treatment for COVID-19, Randomized clinical trial

## Abstract

The COVID-19 pandemic has driven the search for alternative therapies, including convalescent plasma, historically used in infectious diseases. Despite results in other diseases, its effectiveness against COVID-19 remains uncertain with conflicting results in clinical trials. A pragmatic, single-center, prospective, and open randomized controlled trial was carried out in a hospital in Brazil, with the aim of evaluating the impact of convalescent plasma on the clinical improvement of patients hospitalized with COVID-19. The World Health Organization (WHO) ordinal scale was used to measure clinical improvement, focusing on the reduction in disease severity by up to 2 points, while antibody and C-reactive protein levels were monitored over time. After hospital admission, participants were randomized 1:1 to receive convalescent plasma and standard treatment or to be part of the control group with standard treatment. Follow-up was carried out on days 1, 3, 7, 14 and/or at discharge. From January 14 to April 4, 2022, 38 patients were included, but 3 were excluded due to protocol deviations, resulting in a total of 35 patients: 19 in the control group and 16 in the plasma group. There was no significant difference in clinical improvement between the convalescent plasma group and the control group, nor in secondary outcomes. The study had limitations due to the small number of patients and limited representation of COVID-19 cases. Broader investigations are needed to integrate therapies into medical protocols, both for COVID-19 and other diseases. Conducting randomized studies is challenging due to the complexity of medical conditions and the variety of treatments available.

## Introduction

Since its beginning in December 2019, the COVID-19 (Coronavirus Disease 2019) pandemic has infected approximately 772 million people worldwide and caused the deaths of over 6.9 million individuals, with over 13 billion vaccine doses administered. In Brazil, there have been over 37 million cases and around 702 thousand deaths. Since the introduction of COVID-19 vaccines in the country, over 517 thousand doses have been administered, according to data from the WHO (1). Most infected individuals experienced either asymptomatic or mild illness, while some progressed to more severe conditions such as Severe Acute Respiratory Syndrome (SARS). Various therapeutic strategies, including anticoagulants, antibacterial and antiviral agents, corticosteroids, and immunotherapy, have been tested to prevent critical illness and lethality (1).

A traditional approach employed as passive immunotherapy for the treatment of infectious diseases is the use of convalescent plasma obtained from a donor that survived the infection. For influenza A-H1N1 patients, treatment with convalescent plasma significantly reduced mortality and decreased the viral load with no observed adverse events (2,3). Furthermore, a meta-analysis by Mair-Jenkins et al. (4) showed that convalescent plasma administered early after the onset of symptoms may reduce mortality and appears to be safe in patients with severe acute respiratory infections. One possible explanation for the effectiveness of convalescent plasma therapy is that antibodies can suppress viremia, not only limited to eliminating free viruses and blocking new infections, but also to accelerating the viral clearance from the infected cell (5).

Severe acute respiratory syndrome coronavirus 2 (SARS-CoV2), the etiologic agent of COVID-19, infects cells through the engagement of the viral surface spike protein to cellular receptors, of which angiotensin-converting enzyme 2 (ACE2) is the most important (6). As a prominently exposed protein in the viral surface, the spike protein is a major immunogen of SARS-CoV2 (7). Because the SARS-CoV2 spike protein is essential for viral cellular attachment and invasion, huge efforts have been directed to research and development of neutralizing antibodies targeting this protein (8). In this context, the use of COVID-19 convalescent plasma, which carries a mixture of antibodies of different spike epitope specificity, emerges as a therapeutic alternative against SARS-CoV2 (7,9,10). It has been suggested that the use of COVID-19 convalescent plasma contributes to anti-inflammatory, antithrombotic, and immunomodulatory actions that would reduce some of the complications or changes induced by SARS-CoV-2 (11).

Following the initial approval of the Emergency Use Authorization of Convalescent Plasma for COVID-19 infection to treat hospitalized patients in August 2020 by the US Food and Drug Administration (FDA), many clinical trials have been conducted. Some of them reported no benefits, such as the RECOVERY trial that transfused plasma bags containing minimum titers of 1:100 to hospitalized patients with a median time from symptom onset of 9 days. An Argentinian trial employed convalescent plasma with titers greater than 1:1000 to treat older non-hospitalized adults within 72 h after the onset of mild COVID symptoms and reported a 48% relative risk reduction of severe respiratory disease development (12,13). Different study designs, heterogeneous populations, different outcomes considered, lack of standardization of antibody titers in the plasma employed, the time of plasma administration, and the recipient's immunological status are factors that hinder study comparisons and could be the cause of the discrepancies between the results (14,15).

Given the barriers to global access to expensive therapies, such as monoclonal antibodies and antiviral agents, and the rise of antibody resistance, convalescent plasma arose as a possible treatment approach available even in resource-deprived areas of the world. Despite numerous clinical trials employing convalescent plasma to treat COVID-19, its usefulness for the disease remains uncertain. This study aimed to evaluate the impact of convalescent plasma on clinical improvement of patients hospitalized with COVID-19. The main evaluation metric was clinical improvement, measured with the WHO ordinal scale, focusing on reduction of disease severity by up to 2 points. Furthermore, antibody levels and C-reactive protein were monitored in relation to days of hospitalization and disease progression.

## Material and Methods

This was a pragmatic, single-center, prospective, open-label, randomized controlled institutional standard care study. The study took place from March 2021 to April 2022 and was carried out at the Maternidade e Cirurgia Nossa Senhora do Rocio, located in the municipality of Campo Largo, Paraná, Brazil, with patients screened after admission for hospitalization. Informed consent was obtained from all participants and the study was conducted in accordance with the principles of the Declaration of Helsinki and the Good Clinical Practice guidelines, as authorized by the Ethics and Research Committee (No. 46600921.5.0000.5225). The authors take full responsibility for the design and conduct of the study and ensure the accuracy and integrity of the data, data analysis, and adherence to the study protocol.

### Inclusion and exclusion criteria

The inclusion criteria were hospitalized adults aged 18 years or older with a COVID-19 diagnosis or exacerbation, positive RT-PCR test or SARS-CoV-2 antigen in a respiratory tract sample, duration of hospitalization of 5 consecutive days or less, up to 7 days of onset of signs and symptoms of the disease, and at risk of severity according to the institutional classification: mild, with signs and symptoms of COVID-19 but without shortness of breath, dyspnea, or abnormal chest images; moderate, with signs of lower respiratory disease during clinical or imaging evaluation and with a SpO_2_ >94% and lung injury of 20 to 50%; or severe, with SpO_2_ <94%, ratio of arterial partial oxygen pressure to fraction of inspired oxygen (PaO_2_/FiO_2_) <300 mm Hg, respiratory rate >30 breaths/min, and lung injury >50%. We excluded participants with physical examination findings, laboratory abnormalities and/or history of any disease that could jeopardize their safety in participating in the study, with evidence of critical COVID-19 and a history of anaphylactic reaction related to blood component transfusion.

Regarding comorbidity factor, those patients who reported the existence of the disease with previous diagnoses and treatment were considered. All protocols used were institutional standards.

### Randomization and intervention

Randomization was performed in blocks using the secure, web-based, and automated randomization system Research Electronic Data Capture, RedCap (version 11.0.3, USA).

The randomization involved the random allocation of individuals into groups (blocks) of fixed size. Within each block, individuals were randomly distributed, thus ensuring the absence of bias in the allocation of participants, as well as a balanced distribution.

Participants were allocated in a 1:1 ratio. The plasma group could receive up to 2 bags of convalescent plasma of approximately 200 to 300 mL each according to the institution's transfusion standard and after pre-transfusion exams, in addition to the institutional standard of care. The control group received the standard treatment of the institution, including antivirals, antibiotics, steroids, and O_2_ when necessary.

Study patients were monitored on days 1, 3, 7, and 14 and/or hospital discharge by the assistant professional using a data collection instrument. If the outcome of the patients was discharge due to cure, on request, transfer to another center, or death, monitoring would occur until the moment the patient left the institution.

### Convalescent plasma donation

Donor recruitment took place through campaigns on social media calling for individuals who had COVID-19 infection proven through laboratory tests, with an interval of more than 30 days from diagnosis, who had not received blood transfusion or needed mechanical ventilation, and who were male.

The entire process was carried out by the Hematology and Hemotherapy Center of Paraná. The lowest titer accepted for the study was an index of 66.18 U/mL. Convalescent plasma bags were from a single donor or from a pool of two to five donors.

### Clinical outcomes

Outcomes were assessed based on the WHO clinical case classification for COVID-19: 1) Outpatients with resumption of normal activities; 2) Outpatients but unable to resume normal activities; 3) Hospitalized, without the need for oxygen supplementation; 4) Hospitalized, needing supplemental oxygen; 5) Hospitalized, requiring high-flow nasal oxygen therapy, non-invasive mechanical ventilation, or both; 6) Hospitalized, requiring extracorporeal membrane oxygenation (ECMO), invasive mechanical ventilation or both; and 7) Death. The other analyses were based on clinical status on the ordinal scale on the 14th day or hospital discharge with improvement of 2 and 3 points on the scale, antibody titers in relation to the days of hospitalization, and C-reactive protein assessed based on the days of disease progression. C-reactive protein was used as a marker of inflammation and severity, where high levels may indicate acute inflammation, suggesting a more serious infection. To evaluate C-reactive protein levels, the turbidimetry methodology was used, with a reference value of <10.0 mg/L.

For statistical analysis, the association between convalescent plasma and control with the ordinal primary outcome of the WHO scale was evaluated using an ordinal logistic regression model with generalized estimating equations (GEE) with interchangeable correlation matrix and adjustment for baseline values, gender, age, and history of chronic obstructive pulmonary disease (COPD). The main focus of the analysis was improvement at hospital discharge compared to the first day of treatment. The results are reported as a function of the proportional odds ratios for the ordinal categories and their respective 95% confidence intervals.

Spearman's partial correlation coefficient was used to verify the effect of titer and days of hospitalization on the improvement in the scale. P<0.05 was considered significant. Statistical analysis was performed using SAS v9.4 (USA). This study is registered at ClinicalTrials.gov, NCT05077930.

## Results

**Figure 1:**
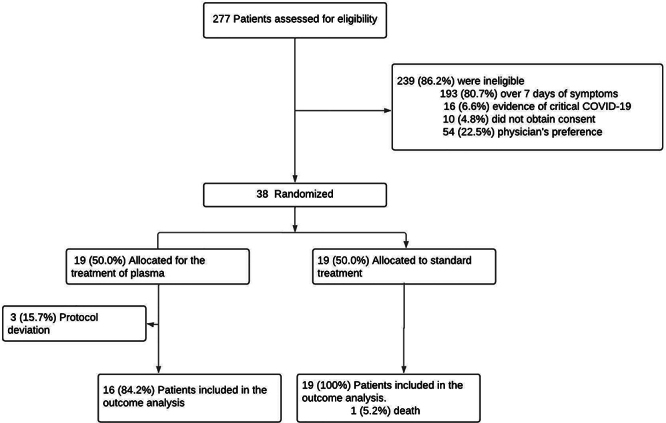
Trial flow chart, participant enrollment, and randomization.

In the study period from January 14 to April 4, 2022, a total of 277 patients were screened for the eligibility criteria during hospitalization; 38 patients who met the inclusion criteria were enrolled. Participants were randomized in a 1:1 ratio, totalizing 19 patients in the group that received the institutional standard treatment (control) and 19 patients that received convalescent plasma and institutional standard treatment. In the latter, however, 3 patients were considered as protocol deviations, totaling 35 patients evaluated and monitored in the study (Figure 1).

In the analysis of the epidemiological and clinical characteristics of the study patients, the difference in mean age between the control and plasma groups was not significant, with an average of 68 years for the former and 59 years for the latter. Gender distribution was balanced in both groups, with 51% of patients being male. Homogeneous gender distribution is relevant when considering possible gender differences in treatment response and clinical outcomes.

The length of hospitalization and the onset of symptoms were similar in both groups, with the majority of patients presenting up to 2 days of hospitalization and 4 days of onset of symptoms. This uniformity in hospitalization time and symptom onset suggests homogeneity in disease severity and stage of disease progression between groups, which is fundamental when comparing treatment results.

The majority of cases were classified as mild in both groups, with 84.21% of patients in the control group and 81.25% of patients in the plasma group being classified as mild.

All patients in the control group were vaccinated, while in the plasma group, 87.50% of patients had received the vaccine (Table 1).

**Table 1:** Epidemiological and clinical characteristics of COVID-19 patients.

Characteristic	Control (n=19)	Plasma (n=16)	Overall (n=35)	P-value
Mean age, in years	68.05±13.30	59.25±17.56	64.03±15.79	0.10
Gender				0.87
Male	10 (52.63)	8 (50.00)	18 (51.43)	
Female	9 (47.37)	8 (50.00)	17 (48.57)	
Ethnicity				0.42
White	15 (78.94)	11 (68.75)	26 (76.28)	
Black	0 (0.00)	1 (6.25)	1 (2.85)	
Brown	2 (10.52)	1 (6.25)	3 (8.57)	
Unknown	2 (10.52)	3 (18.75)	5(20.00)	
Exam				1.00
RT-PCR	3 (15.79)	2 (12.50)	5 (14.29)	
Serology	16 (84.21)	14 (87.50)	30 (85.71)	
Mean days of hospitalization	1.89±0.88	2.00±1.26	1.94±1.06	0.94
Hospitalization				0.60
1 day	8 (42.11)	8 (50.00)	16 (45.71)	
2 days	5 (26.32)	3 (18.75)	8 (22.86)	
3 days	6 (31.58)	3 (18.75)	9 (25.71)	
4 days	0 (0.00)	1 (6.25)	1 (2.86)	
5 days	0 (0.00)	1 (6.25)	1 (2.86)	
Mean days with signs and symptoms	4.53±1.43	4.88±1.89	4.69±1.64	0.45
Signs and symptoms				0.22
2 days	1 (5.26)	3 (18.75)	4 (11.43)	
3 days	5 (26.32)	1 (6.25)	6 (17.14)	
4 days	3 (15.79)	3 (18.75)	6 (17.14)	
5 days	4 (21.05)	1 (6.25)	5 (14.29)	
6 days	5 (26.32)	4 (25.00)	9 (25.71)	
7 days	1 (5.26)	4 (25.00)	5 (14.29)	
COVID-19 severity classification				0.80
Mild	16 (84.21)	13 (81.25)	29 (82.86)	
Moderate	3 (15.79)	2 (12.50)	5 (14.29)	
Severe	0 (0.00)	1 (6.25)	1 (2.86)	
Chronic disease				0.67
Yes	12 (63.16)	9 (56.25)	21 (60.00)	
No	7 (36.84)	7 (43.75)	14 (40.00)	
Hypertension				0.47
Yes	14 (73.68)	10 (62.50)	24 (68.57)	
No	5 (26.32)	6 (37.50)	11 (31.43)	
Chronic obstructive pulmonary disease				1.00
Yes	4 (21.05)	4 (25.00)	8 (22.86)	
No	15 (78.95)	12 (75.00)	27 (77.14)	
Autoimmune disease				0.44
Yes	0 (0.00)	1 (6.25)	1 (2.94)	
No	19 (100.00)	15 (93.75)	33 (97.06)	
Diabetes mellitus				0.71
Yes	6 (31.58)	6 (37.50)	12 (34.29)	
No	13 (68.42)	10 (62.50)	23 (65.71)	
Vaccinated for COVID-19				0.20
Yes	19 (100.00)	14 (87.50)	33 (94.29)	
No	0 (0.00)	2 (12.50)	2 (5.71)	

Categorical data are reported as number (percentage) and continuous variables as means±SD. P-value calculated by chi-squared, exact chi-squared, or Mann-Whitney tests.

At hospital discharge, compared to the first day of treatment, no significant difference was observed between the convalescent plasma group and the control group in patient improvement according to the ordinal scale (OR=1.95; 95% confidence interval 0.35 to 10.91; P=0.4487). After adjusting for gender, age, and COPD history, the odds ratio for patient improvement between the convalescent plasma and placebo groups was 2.32 (95% range 0.29 to 18.51; P=0.4267), remaining non-significant. There was no difference in clinical improvement between groups (Table 2).

**Table 2:** Percentage of improvement on the World Health Organization (WHO) scale (in points) according to type of treatment.

WHO Scale - Improvement at discharge compared to the 1st day of treatment	Group		Not Adjusted (Plasma × Control)		Adjusted (Plasma × Control)^#^	
	Control	Plasma	OR (95%CI)	P-value	OR (95%CI)	P-value
Scale improvement (in points)			1.95 (0.35; 10.91)	0.44	2.32 (0.29; 18.51)	0.42
2 points (%)	77.78±9.95	73.33±11.59				
3 points (%)	22.22±9.95	26.67±11.59				

Data are reported as mean±SE. ^#^Adjusted for sex, age, and chronic obstructive pulmonary disease. P-values were calculated using an ordinal logistic regression model with generalized estimating equations.

The median volume of convalescent plasma infused was 250 mL, with an interquartile range from 200 to 300 mL. This consistency in the infused volume suggests uniformity in the treatment protocol adopted, following standardization and effectiveness of the procedure in administering an adequate amount of plasma to patients. All patients received only one bag of convalescent plasma during the infusion procedure.

At infusion, the average titer of total antibodies and neutralizing antibodies to SARS-COV-2 was 209 U/mL, with a range from 66.18 to 1,648.55 U/mL. This wide variation in antibody concentration may reflect individual differences in patients' immune response or variations in the quality of the convalescent plasma used.

Regarding correlations between improvement in the WHO scale, convalescent plasma antibody titer, and days of hospitalization, direct but non-significant correlations were observed (r=0.44; P=0.0779 and r=0.32; P=0.2174, respectively).

Partial correlation of improvement in the WHO scale with antibody titer and days of hospitalization changed in relation to the simple correlations but remained direct and not significant (r=0.32; P=0.2319 and r=0.04; P=0.8931, respectively). Even after controlling for possible confounding factors, such as gender, age, or clinical history, a statistically significant relationship was not found between the patients' clinical improvement and the analyzed variables (Table 3).

**Table 3:** Crude and partial Spearman correlation coefficients of titer and days of hospitalization with improvement in the scale.

Scale improvement	Spearman correlation coefficient (95%CI)	P-value	Spearman partial correlation coefficient (95%CI)	P-value
Improved scale				
Titration	0.44 (-0.07; 0.77)	0.07	0.32 (-0.23; 0.72)	0.23
Days of hospitalization	0.32 (-0.21; 0.70)	0.21	0.04 (-0.48; 0.54)	0.89

When analyzing the change in C-reactive protein on the logarithmic scale on the third day in relation to the first day of treatment, it was found that there was no significant difference between treatments (P=0.5367). Similarly, the change on the seventh day in relation to the first day of treatment was not significantly different between the groups (P=0.7065).

Regarding mean C-reactive protein values on the logarithmic scale throughout the follow-up period, no significant differences were observed between treatments (P=0.8097) (Table 4).

**Table 4:** Average C-reactive protein values (on a logarithmic scale) according to type of treatment.

	Treatment		P-value^#^	Change compared to the 1st day P-value
	Control	Plasma	Interaction between treatment and time	Plasma × Control
C-reactive protein (on logarithmic scale)			0.80	
1st day	3.23±0.28	2.91±0.31		-
3rd day	3.07±0.34	2.44±0.40		0.53
7th day	2.82±0.51	2.23±0.52		0.70

Data are reported as mean±SE. ^#^P-value obtained from fitting the linear mixed effects model.

## Discussion

In this study, we found that patients receiving the usual care plus convalescent plasma had no significant improvement in clinical outcomes according to the ordinal WHO scale compared to those who received only the usual care. There was also no evidence of benefit in the convalescent plasma group for any of the pre-specified secondary endpoints.

Through systematic reviews on various viral infections, such as different types of influenza (H1N1, H5N1, among others), MERS-CoV, SARS-CoV, and Ebola, it was observed that the results of using convalescent plasma in improving mortality are conflicting (4,16) or show no benefit (17). Most studies that show improvement in mortality have a moderate to high risk of bias linked to the absence of randomization of patients, and most of the studies were not blinded and do not have a comparison group (4). Due to the non-standardization of the methodology applied, including the titers of the convalescent plasma in a very heterogeneous population, it is difficult to compare and reach a conclusion about the use of convalescent plasma, which requires more well-standardized studies.

Conflicting results were also found when evaluating the use of convalescent plasma in COVID-19 patients. The RECOVERY, CONCOR-1, and REMAP-CAP trials found no benefit for high titer convalescent plasma therapy in hospitalized patients and were stopped early due to futility. CSSC-004 was a double-blind, placebo-controlled trial evaluating treatment with convalescent plasma in adults with less than 8 days of COVID-19 symptoms, where there was a reduction in the proportion of patients who experienced COVID-19-related hospitalizations within 28 days (2.9 *vs* 6.3% in the placebo arm). SIREN-C3PO and CONV-ERT trials found no benefits of convalescent plasma treatment in non-hospitalized COVID-19 patients with ≤7 days of mild and moderate symptoms based on the proportion of patients who experienced disease progression, were hospitalized, or died. Such results must be interpreted with caution due to different administration time points, antibody titer, patient populations, convalescent plasma manufacturing process, control arms considered, and outcomes evaluated. Antagonistic results are also found in systematic reviews as a result of available data at that specific moment and endpoints evaluated.

The convalescent plasma therapy aims to generate transient passive immunity in the recipient to control the active infection. However, the temporal principle is one of the basic principles of antibody therapy and states that antibody preparations are most effective when administered prophylactically or early in the course of the disease. Unfortunately, such principles were not considered during the early days of the COVID-19 pandemic, and convalescent plasma therapy was used primarily as a salvage therapy in severe disease when other therapies were ineffective (18).

It is important to highlight that the collection and treatment of convalescent plasma were carried out in two very different scenarios of infection by the SARS-CoV2 variant in Brazil.

Since the initial isolation of SARS-CoV-2, genomic analyses have revealed that the virus has evolved into multiple variants with mutations that increased its transmissibility, infectivity, and immune evasion ability (19). Prominent variants include Alpha (B.1.1.7), Beta (B.1.351), Gamma (P.1), Kappa (B.1.617.1), Delta (B.1.617.2), and Omicron (B.1.1.529), and their sub lineages. The Omicron variant has emerged as globally dominant (20), characterized by more than 30 changes in the receptor-binding domain (RBD) of the spike protein, conferring resistance to many known neutralizing antibodies (21,22).

During the study period (January to April, 2022), the predominant variants in Brazil were Delta and especially Omicron (BA.1, BA.1.1, BA.1., BA.2, BA.2.) (23). However, convalescent plasma samples were collected in June 2021, when the variability of variants was high, including Gamma, Alpha, Beta, and Delta (23). The Omicron variant showed significant resistance to neutralization by monoclonal antibodies and convalescent plasma from patients infected with previous variants such as WA-1, Alpha, Beta, Delta, and Gamma (24).

Thus, plasma from Gamma convalescent donors shows a 12-fold reduction in neutralizing potency against the Omicron variant compared to the Gamma variant (24). Therefore, we postulated that the lack of effect of convalescent plasma treatment in our study could be largely due to immunological escape derived from different variants, with the time difference between the application of convalescent plasma in patients and its collection being responsible for generating a limitation in terms of neutralizing antibodies due to the presence of different variants.

As of December 2021, the FDA has approved the use of COVID-19 convalescent plasma with high levels of anti-SARS-CoV2 antibodies only in patients who have immunosuppressive disease or who are receiving immunosuppressive treatment. Furthermore, NIH guidelines recommend that the plasma used must have been collected after the emergence of the Omicron variant (25,26). It is worth noting that these guidelines are continually updated based on the emergence of new results.

At the beginning of this study, we planned to recruit 200 subjects, but only 38 patients were enrolled, ending up with 35 patients, which decreased the study power. This happened because, over time, fewer individuals sought medical care due to COVID-19 symptoms, probably as a result of vaccination. Vaccination has numerous advantages in controlling outbreaks, but we must not forget that there is inequality in the distribution and access to vaccines across the world. Different legal, economic, social, and demographic issues generate a vulnerable population that still needs cheap treatment strategies to treat COVID-19 (27). In this sense, continuous studies with convalescent plasma therapy become important.

Antibody titers in plasma sacs are one of the most important factors in convalescent plasma therapy. In our study, we found variability in the concentration of antibodies infused, which could be attributed to individual differences in the patients' immunological response or to variations in the quality of the convalescent plasma used. Libster et al. (13) observed a dose-dependent effect for SARS-CoV2 S IgG titers in convalescent plasma used to treat elderly patients with COVID-19. Median plasma titers of 1:3200 induced a 73% relative risk reduction of worsening disease. We were also unable to find a relationship between antibody titer and number of days of hospitalization, even after controlling for possible confounding factors, such as gender, age or clinical history.

Another crucial point in determining the potency and potential harm of convalescent plasma therapy is its antibody content (28). CONCOR-1, a multicenter, open-label, randomized trial of 921 patients, reported that an increase in neutralization or antibody-dependent cellular cytotoxicity reduced the harmful effect of convalescent plasma. On the other hand, increased levels of IgG against the total transmembrane spike protein were associated with a deleterious effect on the primary outcome, which was intubation or death within 30 days. Unfortunately, we did not have data on antibody content in the plasma bags used in this study, but considering that patients treated with convalescent plasma did not have worse results, we assumed that this was not a major problem.

The biochemical monitoring of patients affected by SARS-CoV-2 was extremely important for assessing the severity and evolution of the disease. Several studies have investigated the profile of laboratory markers in patients hospitalized due to COVID-19. In the study by Wang et al., it is suggested that the risk of developing serious events increases by 5% for each increased unit in the concentration of C-reactive protein in patients with COVID-19 (29), whereas in this study, when analyzing C-reactive protein values on the logarithmic scale throughout the follow-up period, no significant differences were identified between treatments. However, it is important to consider that C-reactive protein levels can be influenced by a series of pre-existing conditions, which can lead to variability in results. The lack of significant differences between treatment groups can be interpreted in several ways, providing important insights into the effectiveness of convalescent plasma. One possible interpretation is that despite the patients' chronic conditions, convalescent plasma did not have a substantial impact on reducing C-reactive protein levels compared to standard care. It is also plausible that underlying chronic conditions masked any specific treatment effects, making it more difficult to identify significant differences in outcomes.

This study had some limitations, mainly related to the small number of patients recruited and the variability of COVID-19 variants circulating during the study period. In this sense, it is imperative to reevaluate the use of convalescent plasma as a standard treatment, considering the possibility of implementing other therapies, such as intravenous immunoglobulin or anti-SARS-CoV-2 monoclonal antibodies. It is crucial to highlight that validating the effectiveness of these alternatives requires further investigation through clinical studies in different contexts to gain a complete understanding of their effects and applicability.

Conducting broader research will contribute significantly to developing a solid evidence base on convalescent plasma and other therapies, enabling their safe and effective integration into medical treatment protocols, not only for COVID-19, but also for other emerging viral diseases. The difficulty in implementing randomized studies, as observed in this study, is recognized and attributed in part to the complexity of the medical conditions studied and the variety of treatments available, highlighting ethical, logistical, and practical challenges associated with this type of research.
